# Topical probiotic *Lactobacillus lactis* treatment in atopic dermatitis: a placebo-controlled pilot study on tolerability and efficacy

**DOI:** 10.3389/fmed.2026.1694229

**Published:** 2026-02-03

**Authors:** Ville Salo, Anita Remitz, Antti Lauerma, Alexander Salava

**Affiliations:** Department of Dermatology and Allergology, Helsinki University Hospital and University of Helsinki, Helsinki, Finland

**Keywords:** atopic dermatitis, *Lactobacillus lactis*, therapy, tolerability, topical probiotic

## Abstract

**Introduction:**

Microbiome-targeted treatments have been investigated in atopic dermatitis (AD). We aimed to investigate the tolerability and efficacy of probiotic *Lactobacillus lactis* lysate cream in AD.

**Methods:**

A total of 13 patients with mild-to-moderate AD were treated with differently concentrated probiotic creams (3, 10, and 30%) for 4 weeks. The severity of AD [Eczema Area and Severity Index (EASI) and Investigator Global Assessment (IGA)], epidermal barrier function (TEWL), and the impact of AD [Dermatology Life Quality Index (DLQI), Patient-Oriented Eczema Measure (POEM), Atopic Dermatitis Control Tool (ADCT), and pruritus and sleep disturbance visual analog scale (VAS)] were measured at baseline (BL) and at 4 and 8 weeks. Comprehensive clinical patient data and laboratory values, including blood eosinophil count, total serum IgE levels, and specific IgEs to aeroallergens, were obtained.

**Results:**

Comparison of the treatment groups and longitudinal comparisons at various time points showed no significant differences regarding AD severity (EASI, *p* = 0.76, CI: 0.65–1.00), epidermal barrier dysfunction (TEWL, *p* = 0.37, CI: 0.19–0.73), or patient-reported subjective impact of AD (DLQI, *p* = 0.76, CI: 0.65–1.00; POEM, *p* = 0.76, CI: 0.35–0.88; ADCT, *p* = 0.72, CI: 0.65–1.00; pruritus VAS 0.67, CI: 0.55–1.00; sleep disturbance VAS, *p* = 1.00, CI: 0.79–1.00) between different probiotic lysate concentrations and placebo. The probiotic lysate cream was well-tolerated, and there were no significant adverse effects. The limitations of the study were the small patient cohort and group sizes. There was also a relatively short follow-up, and no evaluation of long-term effects was conducted.

**Discussion:**

In our patient cohort, topical probiotic *L. lactis* lysate cream showed good tolerability, but it did not show efficacy in the treatment of mild-to-moderate AD. Although topical probiotics have been reported to be effective in a limited number of studies, more placebo-controlled clinical studies are needed to explore their potential role in the treatment of AD.

**Clinical trial registration:**

https://eudract.ema.europa.eu, Identifier EudraCT 2020-000514-15.

## 1 Introduction

Atopic dermatitis (AD) is one of the most frequent skin disorders in developed countries and affects approximately 10–20% of the adult Finnish population (1). The pathogenesis of AD is still not completely understood, but it has been recognized in recent years that disease onset and flares are caused by an interplay of intrinsic (polygenetic inheritance) and extrinsic triggers (microbes and allergens) (2). Three key components in the pathogenesis have been established: 1. Mutations in genes important for the epidermal barrier function (3, 4) (e.g., filaggrin), 2. Immunological changes (5, 6), such as native and adaptive immune system, atopic diathesis, and susceptibility to skin infections, and 3. Changes in the skin microbiome, for example, the skin colonizing commensal microbes, decrease in bacterial diversity, and colonization by filaggrin-degrading *Staphylococcus aureus* strains (7–9).

There are no curative therapies available, but with appropriate treatment, the symptoms can be managed and remissions can be achieved. Courses of topical corticosteroids or calcineurin inhibitors with concomitant moisturizing emollients are considered first-line treatments. In severe or treatment-recalcitrant cases, systemic immunomodulatory agents or phototherapies have been used. Oral and topical probiotics have also been investigated in AD, but their efficacy remains controversial (10, 11). Probiotics are believed to maintain a healthy microbial flora and to prevent colonization with pathogenic microbes (colonization resistance) and have included parts (lysate) or live microorganisms that do not necessarily have to be part of the patients’ microbiome. In this study, we aimed to compare the efficacy of probiotic *Lactobacillus lactis* lysate cream in different concentrations of AD. We specifically intended to investigate the tolerability and effects of high concentrations of topical probiotic lysate creams.

## 2 Materials and methods

The study was conducted as a prospective, double-blinded, randomized, placebo-controlled, split-body clinical interventional study that was carried out in a single center (Helsinki University Hospital) from September 2021 to May 2022. It was designed as an explorative pilot study with a limited number of participants. Patient recruitment and randomization were carried out in the hospital, and the inclusion criteria were mild-to-moderate AD (according to EASI) (12) and age over 18 years. The exclusion criteria were severe AD, pregnancy or breastfeeding, use of systemic immunomodulatory medication, other concomitant chronic diseases, signs of immune suppression, or participation in another biomedical research during the intervention. The study protocol was approved and registered prior to recruitment by the Finnish Medicines Agency (nr. 20/2020, https://fimea.fi/en/frontpage) and the European Union Drug Regulating Authorities Clinical Trials Database (EudraCT 2020-000514-15, https://eudract.ema.europa.eu/) and was approved by the ethics committee of the Helsinki University Hospital, Finland (HUS/3359/2019) according to the Declaration of Helsinki.

### 2.1 Patients

There were 13 (4 male and 9 female) patients aged 18–56 years (median age: 30 years) with mild-to-moderate AD. All patients provided written consent to participate in the study. Baseline eosinophil counts, total serum IgE levels, specific IgEs to aeroallergens, and comprehensive clinical patient characteristics (e.g., AD onset, previous therapies, need for hospitalization, and atopic comorbidities) are presented in Supplementary material 1.

The patients were randomly assigned to one of the four patient groups:

1. Probiotic lysate 3% cream
2. Probiotic lysate 10% cream
3. Probiotic lysate 30% cream
4. Placebo group (vehicle cream without probiotic *L. lactis* lysate)

Randomization was carried out manually and was based on the double-blinded study setting.

### 2.2 Treatment protocol

The study period included a 1-week washout period prior to the beginning, an 8-week study period, and three doctor’s appointments. During the study, patients were allowed to use basic ointments and were advised to avoid friction and wash their skin once daily with a mild detergent. In case of AD exacerbation, the patients were allowed to use a topical hydrocortisone-17-butyrate cream for courses of 1–2 weeks. Patients were investigated by an experienced dermatologist at baseline, after 4 weeks of topical probiotic lysate treatment, and after 4 weeks of follow-up (8 weeks after baseline). Topical treatment was carried out on one forearm and upper arm (unilaterally, approximately 7.5% of body surface), with the investigated *L. lactis* lysate or placebo cream twice per day (total amount of 250–300 g cream). The contralateral arm was treated according to normal practice. After 4 weeks, the investigated treatment was discontinued, and another follow-up visit was carried out (8 weeks after baseline). During the 4 weeks of discontinuation of topical probiotic treatment, the skin was treated according to normal practice.

During each doctor’s appointment, the patient’s whole skin was evaluated, and the severity of AD was characterized with investigator-dependent methods (EASI and IGA). Additionally, the epidermal barrier function was measured (transepidermal water loss, TEWL) (13). Furthermore, EASI was divided into local and total EASI, in which “local” measured only the affected upper limb on which the topical treatment was carried out and “total” measured the whole body. TEWL was measured from the eczema-affected site on the affected upper limb on which the topical treatment was carried out and from healthy skin from the opposite limb.

Patient-reported AD severity and QoL data, such as pruritus and sleep disturbance VAS, DLQI, POEM, and ADCT, were also obtained. Blood samples were obtained from each patient during the baseline visit for blood eosinophil count, total serum IgE levels, and specific IgEs to aeroallergens such as birch, timothy, mugwort, cat, dog, horse, *Cladosporium herbarum*, and *Dermatophagoides pteronyssinus*. The study protocol is presented in Supplementary material 2.

### 2.3 Probiotic *L. lactis* lysate cream

The investigated topical treatment (probiotic *L. lactis* lysate cream) was an oil-in-water emulsion (cream) with different concentrations of the investigated probiotic lysate. The different probiotic lysate concentrations were 3, 10, and 30%. The placebo cream did not include any probiotic lysate and consisted only of the vehicle cream. The vehicle cream consisted of the following components: shea butter, isopropyl palmitate, medium-chain triglycerides, Emulcire 61 (cetyl alcohol, ceteth-20, and steareth-20), Gelot 64 (glyceryl stearate and PEG-75 stearate), glycerol, 1,3-butylene glycol, xanthan gum, purified water, and sodium lactate. The experimental creams contained *L. lactis* lysate. The composition of vehicle and experimental creams is also presented in Supplementary material 3: Table 5. Vehicle and probiotic lysate creams were provided by Orion Pharma Ltd., Finland.

### 2.4 Statistical analysis

Dependency between numeric and ordinal variables was calculated using the Kruskal–Wallis *H*-test to compare different patient groups against one another at different time points (baseline, 4 weeks, and 8 weeks) of the study protocol. Comparison was carried out within the same groups at different time points of the study using the Friedman test. A *p*-value of less than 0.05 was considered significant. Statistical analyses were performed using IBM SPSS Statistics for Windows, Version 27.0. Armonk, NY: IBM Corp., USA.

## 3 Results

**Figure 1:**
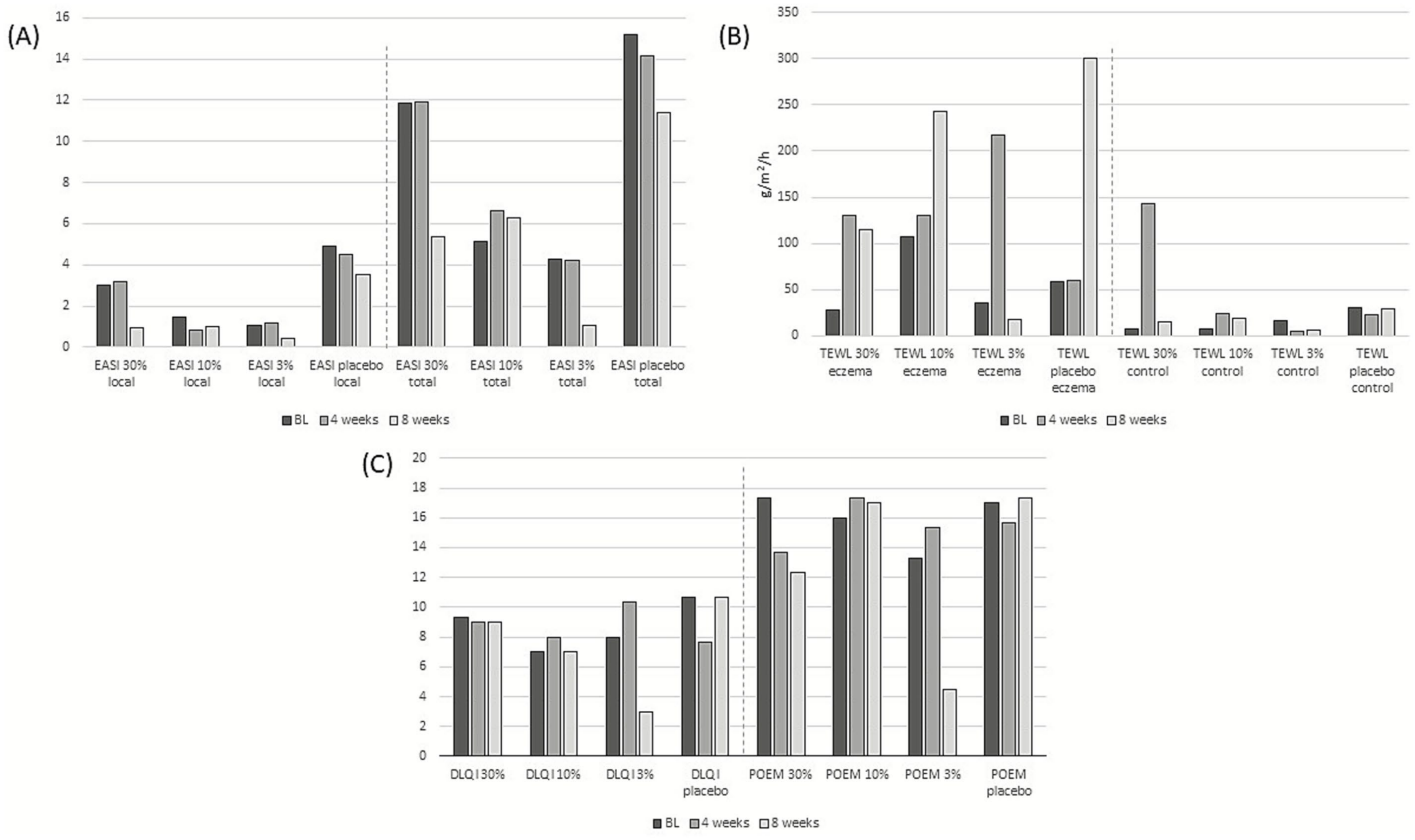
Mean changes in **(A)** EASI local and total, **(B)** TEWL eczema and control, and **(C)** quality of life indicators (DLQI and POEM) over the 8-week study period.

There were no significant differences between the treatment groups regarding disease severity, as assessed by EASI and IGA, TEWL, or patient-reported QoL-associated parameters, including pruritus and sleep disturbance VAS scores, DLQI, POEM, and ADCT. The mean changes in selected disease-severity (EASI local and total), TEWL, and QoL indicators, such as DLQI and POEM, over the 8-week study period are shown in Figure 1, and complete results are presented in Supplementary material 3: Tables 1–4.

### 3.1 Disease severity

The mean local (eczema site) EASI baseline values were 3.00, 1.47, and 1.05 in the probiotic lysate groups and 4.93 in the placebo group. The baseline values of disease severity were higher in the placebo group, probably due to randomization. After 4 weeks of topical treatment, local EASI values were 0.93, 1.00, and 0.40 in the probiotic lysate groups and 3.53 in the placebo group, with no significant decrease compared to baseline (*p* = 0.76). Total EASI values showed similar observations (*p* = 0.31), and there were also no differences between local EASI and total EASI in the patient groups. The IGA values showed similar results (*p* = 0.61), with no statistically significant changes during baseline, 4 weeks of treatment, and 4 weeks of follow-up time. The results regarding AD severity are presented in Table 1.

**Table 1:** Severity of atopic dermatitis and TEWL values and the results of follow-ups and comparisons.

AD severity and TEWL	30% probiotic lysate	10% probiotic lysate	3% probiotic lysate	Placebo	Comparison 30% vs. placebo^a^	Comparison 30% weeks 0, 4, and 8^b^
EASI BL (local) mean (median, range)	3.00 (2.40, 0.60–6.00)	1.47 (0.60, 0.60–3.20)	1.05 (1.10, 0,40-1,60)	4.93 (1.60, 1.20–12.00)	0.83 (CI: 0.79–1.00)	0.31 (CI: 0.06–0.56)
EASI 4 weeks (local)	3.20 (1.60, 0.00–8.00)	0.80 (0.80, 0.40–1.20)	1.20 (1.00, 0.20–2.40)	4.53 (1.00, 0.60–12.00)	0.83 (CI: 0.79–1.00)	
EASI 8 weeks (local)	0.93 (0.80, 0.00–2.00)	1.00 (0.80, 0.60–1.60)	0.40 (0.40, 0.00–0.80)	3.53 (2.20, 0.40–8.00)	0.28 (CI: 0.12–0.65)	
EASI BL (total)	11.83 (6.60, 0.60–28.30)	5.14 (4.80, 2.90–7.70)	4.25 (4.05, 2.70–6.20)	15.20 (4.80, 2.20–38.60)	0.83 (CI: 0.79–1.00)	0.76 (CI: 0.65–1.00)
EASI 4 weeks (total)	11.93 (4.00, 0.00–31.80)	6.63 (5.70, 2.00–12.20)	4.20 (4.10, 1.40–7.10)	14.17, (6.40, 1.10–35.00)	0.51 (CI: 0.35–0.88)	
EASI 8 weeks (total)	5.37 (6.70, 0.00–9.40)	6.30 (4.50, 1.40–13.00)	1.05 (1.05, 0.50–1.60)	11.37 (4.80, 3.90–25.40)	0.83 (CI: 0.79–1.00)	
IGA BL	1.33 (2.00, 0.00–0.20)	1.00 (1.00, 1.00–1.00)	1.25 (1.00, 1.00–2.00)	1.67 (1.00, 1.00–3.00)	0.82 (CI: 0.79–1.00)	0.61 (CI: 0.37–1.00)
IGA 4 weeks	1.67 (2.00, 0.00–3.00)	1.00 (1.00, 1.00–1.00)	1.00 (1.00, 0.00–2.00)	1.67 (1.00, 1.00–3.00)	1.00 (CI: 0.79–1.00)	
IGA 8 weeks	1.67 (2.00, 0.00–3.00)	1.33 (1.00, 1.00–2.00)	0.50 (0.50, 0.00–1.00)	2.00 (2.00, 1.00–3.00)	0.82 (CI: 0.79–1.00)	
TEWL BL (eczema)	27.73 (18.15, 12.00–53.10)	107.55 (51.85, 32.80–238.00)	35.33 (34.70, 12.00–60.00)	59.18 (51.20, 43.60–82.80)	0.28 (CI: 0.12–0.65)	0.26 (CI: 0.12–0.65)
TEWL 4 weeks (eczema)	130.10 (42.75, 21.10–326.50)	130.35 (94.80, 42.80–253 50)	217.13 (14.40, 2.50–634.50)	60.52 (66.00, 29.80–85.80)	0.83 (CI: 0.79–1.00)	
TEWL 8 weeks (eczema)	114.67 (27.50, 14.00–302.50)	242.90 (53.20, 25.50–650.00)	17.13 (17.13, 4.10–30.20)	300.98 (42.35, 22.60–838.00)	0.51 (CI: 0.44–0.94)	
TEWL BL (control)	7.10 (7.90, 4.10–9.30)	7.98 (8.35, 2.40–13.30)	16.79 (5.33, 2.10–54.40)	30.78 (18.80, 11.50–62.10)	0.05 (CI: 0.00–0.22)	0.37 (CI: 0.19–0.73)
TEWL 4 weeks (control)	143.33 (2.80, 1.70–425.50)	24.10 (8.55, 8.50–55.30)	5.57 (6.56, 2.60–7.60)	23.43 (8.50, 6.60–55.30)	0.51 (CI: 0.44–0.94)	
TEWL 8 weeks (control)	14.60 (15.70, 11.40–16.70)	18.83 (9.10, 5.70–41.70)	5.95 (5.95, 4.90–7.10)	29.23 (18.50, 12.70–56.50)	0.28 (CI: 0.12–0.65)	

a Kruskal–Wallis *H*-test, the rest of the analysis is presented in Supplementary material 3: Tables 1, 2. Comparisons are presented as *p*-values; <0.05 was considered significant.

b Friedman test, the rest of the analysis is presented in Supplementary material 3: Tables 3, 4.

EASI local: calculated from the upper limb of one side (treated with topical probiotic cream or placebo), EASI total: calculated for the whole skin.

TEWL eczema: measured from the upper limb of one side (treated with topical probiotic cream or placebo), TEWL control: measured from the upper limb, which was not treated with experimental cream, unit of TEWL measurement: g/m^2^/h.

BL, baseline; EASI, eczema area and severity index; IGA, Investigator Global Assessment; TEWL, transepidermal water loss.

The values are indicated as follows: mean (median, range).

### 3.2 TEWL, eczema site, and healthy skin

At baseline, the mean TEWL values at eczema sites were 27.73 g/m^2^/h, 107.55 g/m^2^/h, and 35.33 g/m^2^/h in the probiotic lysate groups and 59.18 g/m^2^/h in the placebo group. There were significant changes within the groups during topical treatment (*p* = 0.26). In addition, no statistically significant difference was observed in TEWL at baseline, 4 weeks, and 8 weeks when comparing eczema and contralateral sites. The results are presented in Table 1.

### 3.3 Quality of life, pruritus VAS, and sleep disturbance VAS

QoL-associated parameters did not change significantly during the study period and were similar in all patient groups. The mean DLQI values were 9.33, 7.00, and 8.00 in the probiotic lysate groups and 10.67 in the placebo group. At 4 weeks of intervention, the values were similar: 9.00, 8.00, and 10.33 in the probiotic lysate groups and 7.67 in the placebo group, with no significant differences (*p* = 0.76). POEM and ADCT parameters showed similar values with no significant differences (*p* = 0.76 and *p* = 0.72, respectively). The results are presented in Table 2.

**Table 2:** Atopic dermatitis-induced pruritus, sleep disturbance, and QoL indicators (DLQI, POEM, and ADCT), and the results of follow-ups and comparisons.

Pruritus, sleep disturbance and QoL indicators	30% probiotic lysate	10% probiotic lysate	3% probiotic lysate	Placebo	Comparison 30% vs. placebo^a^	Comparison 30% weeks 0, 4, and 8^b^
Pruritus VAS BL mean (median, range)	5.67 (5.00, 4.00–8.00)	4.67 (4.00, 4.00–6.00)	6.50 (6.50, 5.00–8.00)	6.67 (6.00, 4.00–10.00)	0.66 (CI: 0.54–0.99)	0.67 (CI: 0.55–1.00)
Pruritus VAS 4 weeks	5.00 (5.00, 2.00–8.00)	6.00 (6.00, 4.00–8.00)	7.00 (7.00, 4.00–10.00)	6.33 (6.00, 4.00–9.00)	0.51 (CI: 0.35–0.88)	
Pruritus VAS 8 weeks	5.67 (6.00, 3.00–8.00)	7.33 (7.00, 5.00–10.00)	3.50 (3.50, 3.00–4.00)	7.00 (7.00, 6 00–8.00)	0.50 (CI: 0.35–0.88)	
Sleep disturbance VAS BL	2.67 (0.00, 0.00–8.00)	2.00 (2.00, 1.00–3.00)	4.75 (3.50, 2.00–10.00)	4.00 (2.00, 0.00–10.00)	0.49 (CI: 0.35–0.88)	1.00 (CI: 0.79–1.00)
Sleep disturbance VAS 4 weeks	2.67 (0.00, 0.00–8.00)	2.67 (3.00, 1.00–4.00)	7.33 (7.00, 5.00–10.00)	3.00 (2.00, 0.00–7.00)	0.82 (CI: 0.79–1.00)	
Sleep disturbance VAS 8 weeks	4.33 (6.00, 0.00–7.00)	3.67 (4.00, 1.00–6.00)	2.00 (2.00, 2.00–2.00)	3.33 (3.00, 0.00–7.00)	0.82 (CI: 0.79–1.00)	
DLQI BL	9.33 (9.00, 5.00–14.00)	7.00 (6.00, 1.00–14.00)	8.00 (8.00, 4.00–12.00)	10.67 (5.00, 2.00–25.00)	0.66 (CI: 0.35–0.88)	0.76 (CI: 0.65–1.00)
DLQI 4 weeks	9.00 (8.00, 4.00–15.00)	8.00 (11.00, 2.00–11.00)	10.33 (8.00, 8.00–15.00)	7.67 (5.00, 1.00–17.00)	0.83 (CI: 0.79–1.00)	
DLQI 8 weeks	9.00 (11.00, 4.00–12.00)	7.00 (8.00, 1.00–12.00)	3.00 (3.00, 1.00–5.00)	10.67 (7.00, 3.00–22.00)	0.83 (CI: 0.79–1.00)	
POEM BL	17.33 (15.00, 12.00–25.00)	16.00 (18.00, 9.00–21.00)	13.25 (12.00, 6.00–23.00)	17.00 (16.00, 11.00–24.00)	0.83 (CI: 0.79–1.00)	0.76 (CI: 0.35–0.88)
POEM 4 weeks	13.67 (16.00, 4.00–21.00)	17.33 (21.00, 8.00–23.00)	15.33 (16.00, 13.00–17.00)	15.67 (17.00, 6.00–24.00)	0.51 (CI: 0.35–0.88)	
POEM 8 weeks	12.33 (13.00, 4.00–20.00)	17.00 (21.00, 7.00–23.00)	4.50 (4.50, 4.00–5.00)	17.33 (19.00, 5.00–28.00)	0.51 (CI: 0.35–0.88)	
ADCT BL	10.67 (10.00, 2.00–20.00)	9.67 (9.00, 5.00–15.00)	9.00 (9.50, 3.00–14.00)	13.33 (10.00, 7.00–23.00)	0.66 (CI: 0.65–1.00)	0.72 (CI: 0.65–1.00)
ADCT 4 weeks	11.33 (12.00, 3.00–19.00)	9.67 (10.00, 5.00–14.00)	13.67 (13.00, 10.00–18.00)	12.33 (12.00, 4.00–21.00)	0.66 (CI: 0.65–1.00)	
ADCT 8 weeks	10.67 (14.00, 4.00–14.00)	12.67 (15.00, 8.00–15.00)	5.00 (5.00, 4.00–6.00)	12.67 (11.00, 6.00–21.00)	0.83 (CI: 0.79–1.00)	

a Kruskal–Wallis *H*-test, the rest of the analysis is presented in Supplementary material 3: Tables 1, 2.

b Friedman test, the rest of the analysis is presented in Supplementary material 3: Tables 3, 4.

VAS, visual analog scale; BL, baseline; DLQI, Dermatology Life Quality Index; POEM, Patient-Oriented Eczema Measure; ADCT, Atopic Dermatitis Control Tool.

The values are indicated as follows: mean (median, range).

A detailed description of the study results and the full statistical analyses of disease severity and QoL indicators is presented in Supplementary material 3: Tables 1–4.

### 3.4 Clinical data, eosinophil count, total serum IgEs, and specific IgEs to aeroallergens

The results of comprehensive clinical data during the study and eosinophil counts, total serum IgEs, and specific IgEs to aeroallergens at baseline are described in Supplementary material 1.

Two patients dropped out of the study because of the worsening of AD. One patient dropped out during the 4 weeks of topical treatment and another dropped out during 4 weeks of follow-up. We observed no clinically relevant adverse effects in the patient groups.

## 4 Discussion

In this randomized, double-blinded Finnish clinical study, we did not observe significant efficacy of the investigated probiotic *L. lactis* lysate cream in patients with mild-to-moderate AD. In addition, there were no differences between the patient groups concerning different concentrations of *L. lactis* probiotic lysate. AD severity parameters, TEWL, and patient-reported QoL-associated parameters were similar in the placebo group and probiotic lysate groups. The lack of significant differences between the groups may have been due to the low initial disease severity (EASI) and heterogeneity of the patient cohort. However, the patients showed no adverse effects and good tolerability regarding the topical probiotic treatment.

Although topical probiotics have been explored in a limited number of clinical studies in AD, the results have been heterogeneous and conflicting, as have the probiotic formulations used. To date, limited number of randomized double-blinded studies have investigated the efficacy of topical probiotic lysates in AD.

While enteral probiotics have been studied in detail (14–19) and may offer potential for primary prevention, the role of topical probiotics in the treatment of active AD remains unclear. Only a limited number of studies have investigated the use of topical probiotics in AD, further evidence is needed to determine their efficacy, optimal dosing, and specific bacterial strains needed for treatment. In the majority of the published clinical studies, the use of topical probiotics in AD has exhibited significant efficacy, which is contrary to our findings. Previous studies have been conducted using living and dead bacteria as well as using bacterial lysate.

Probiotic bacteria have been applied to the skin either via baths or by mixing bacteria into a base emollient cream. In a German study, Axt-Gadermann et al. (20) observed significant therapeutic responses to probiotic baths, in which a mixture of nine different living bacterial strains was used: *Lactobacillus plantarum*, *Lactobacillus gasseri*, *Lactobacillus rhamnosus*, *Lactobacillus paracasei*, *Lactobacillus johnsonii*, *Lactobacillus reuteri*, *Bifidobacterium longum*, *Bifidobacterium lactis,* and *Streptococcus thermophilus,* divided into two groups of different concentration. A statistically significant reduction of AD severity [Scoring Atopic Dermatitis (SCORAD)] was observed over the 14-day study period.

However, more commonly, the topical distribution of probiotic bacteria or bacterial lysate has been carried out in the form of an emollient cream. Several studies have investigated the use of probiotics in creams with living (21–23) or dead (24) bacteria or bacterial lysates (25). Live strains of *L. reuteri* DSM 17938 (21), *Lactobacillus sakei* probio 65 (22), *S. thermophilus* S244 (23), a heat-treated strain of *L. johnsonii* NCC 533 (24), and a *Vitreoscilla filiformis* (25) lysate were used in these respective studies. Statistically significant decreases were found in clinical parameters, including SCORAD (21, 24, 25) and TEWL (22, 25), as well as in QoL parameters, namely pruritus VAS (22, 25), and sleep disturbance VAS (25). One study also reported a statistically significant increase in stratum corneum ceramide levels (23). In addition, *L. rhamnosus* GG and *L. reuteri* lysates have been found to stimulate the migration and proliferation of keratinocytes (26). Until recently, only two studies have reported results similar to our study, i.e., no statistically significant decrease in SCORAD (23) or IGA (22).

Clinical studies have also revealed that topically used probiotic bacteria improve skin humidity and reduce the intensity of itching, reddening, and peeling of skin in patients with AD (21, 23–25). In addition, topical microbiome transplantation has been studied. Myles et al. (27) used *Roseomonas mucosa* collected from healthy volunteers and transplanted it into patients with AD. The authors observed statistically significant reductions disease severity, as measured by SCORAD, topical steroid use, and *S. aureus* burden.

In addition to ameliorating disease severity, *in vitro* studies have demonstrated that probiotics can suppress cutaneous inflammation and Th2 responses through immunomodulatory mechanisms. Szöllősi et al. (28) reported that *B. longum* extract exerted pro-differentiating effects on human epidermal keratinocytes. We did not observe a decrease in inflammation (EASI local and total) in our patients treated with *L. lactis* probiotic lysate cream. However, we did not investigate systemic or tissue markers of inflammation.

There have been reports on protective effects of probiotics on barrier function and keratinocyte tight junctions. Some *Lactobacillus salivarius* strains have been shown to markedly reduce relocalization of tight-junction proteins in H_2_O_2_-induced barrier-impaired epithelial cells, thus protecting barrier function (29). In addition, Sultana *et al*. observed a strain-dependent augmentation of epidermal barrier function following treatment with *Lactobacillus* and *Bifidobacterium* lysates (30). We measured TEWL in treated and untreated skin but could not observe significant differences in our patients’ groups and the used *L. lactis* probiotic lysate concentrations.

Topical lactobacilli have been reported to modulate the secretion of the Th1/Th2 switch and Treg cell-related cytokines in AD. Neau et al. (31) have shown in an *in vitro* murine model that three Lactobacilli strains exhibited a protective impact on sensitization, with a decrease in allergen-specific IgE, and on allergy, with a decrease in mast cell degranulation. The authors could identify three novel probiotic strains that may be protective against sensitization in mice. In addition, Holowacz et al. (32) showed anti-inflammatory effects of *L. salivarius* and *L. rhamnosus* in skin inflammation in mice. Although we did not investigate inflammatory markers in our patient cohort, it would be interesting in future research to study the anti-inflammatory potential of topical probiotics in AD.

Probiotics, as a therapeutic modality, are considered relatively safe, although there have been some reports of adverse effects (33–36). These adverse effects primarily occurred in cases with enteral administration and in the setting of intensive care and immunocompromised patients. The majority of probiotic preparations are classified as commercial food supplements and are not under the same medicolegal safety surveillance (pharmacovigilance) as their medicinal equivalents, although there are some probiotic medicaments on the market that are considered medicinal products. In our study, the topical probiotic *L. lactis* lysate cream was well-tolerated, and no clinically adverse effects were observed. To provide fast and comprehensive dissemination of our observations, especially safety and tolerability issues, we have published the study results in the form of preprints (all data and abridged version) (37, 38).

### 4.1 Limitations

Important limitations of the study were small patient groups, which were based on limited resources of the single-center setting. Another limitation was the relatively short follow-up duration of 8 weeks and no evaluation of long-term effects. The study was conducted during autumn–spring of 2021–2022, and the time of year might have influenced the results. Patients entered the study at different times, so their baseline skin condition was not comparable to each other.

### 4.2 Conclusion

AD has been linked to microbiome changes, and topical probiotics have been investigated as potential treatment options. In our cohort of mild-to-moderate AD, topical treatment with probiotic *L. lactis* lysate cream did not seem to be effective; however, it was well-tolerated. Further research, especially clinical interventional studies, is needed to investigate the role of topical probiotics in AD and their effects on the cutaneous microbiome. It would be interesting in future research to investigate anti-inflammatory effects, e.g., regulatory T-cell function or transcriptomics, of topical probiotics and the practicability of topical probiotic creams in larger patient cohorts of AD.

## Data Availability

The original contributions presented in the study are included in the article/Supplementary material, further inquiries can be directed to the corresponding author.
