# Supplementary material for: Topical probiotic Lactobacillus lactis treatment in atopic dermatitis: a placebo-controlled pilot study on tolerability and efficacy
Source: Front Med (Lausanne). 2026 Feb 3;13:1694229. doi: 10.3389/fmed.2026.1694229 (PMC12910470; doi:10.3389/fmed.2026.1694229)
Supplement: Supplementary file 1 [file Table_1.docx]

**Supplementary material 1:**

| Patient | Cream | Gender (F/M) | Age | Age of AD onset | Previous topical therapy, phototherapy or systemic medication | Previous need for hospitalization due to AD | Atopic comorbidities | Eosinophiles  (E9/l) | IgE (kU/l) | Aeroallergens (kU/l) | Horse (kU/l) | Cat (kU/l) | Dog (kU/l) | C.herbarum (kU/l) | Birch (kU/l) | Timothy (kU/l) | Common mugwort (kU/l) | House dust mite (kU/l) |
| --- | --- | --- | --- | --- | --- | --- | --- | --- | --- | --- | --- | --- | --- | --- | --- | --- | --- | --- |
| 1 | Placebo | F | 22 | 2 y | TCS, TCI | No | No | 0,26 | 3 | 0,01 | - | - | - | - | - | - | - | - |
| 2 | Placebo | F | 50 | childhood | TCS, TCI | No | No | 0,13 | 121 | 0,05 | - | - | - | - | - | - | - | - |
| 3 | Placebo | F | 56 | childhood | TCS, TCI, CyA, MTX | No | Asthma, ARC |  | 4251 | 6,9 | 1,48 | 2,27 | 2,91 | 2,86 | 0,08 | 8,52 | 0,25 | 0,63 |
| 4 | 3 % | F | 18 | adolescence | TCS, TCI | No | ARC, Food allergy | 0,25 | 738 | 73,4 | 0,08 | 0,82 | 1,2 | 0,04 | 100 | 2,68 | 0,81 | 0,36 |
| 5 | 3 % | F | 23 | childhood | TCS, TCI | No | No | 0,3 | 81 | 0,8 | 0,04 | 1,04 | 0,41 | 0,14 | 0,32 | 0,02 | 0,23 | 0,14 |
| 6 | 3 % | M | 30 | 1 y | TCS, TCI | No | ARC | 0,03 | 558 | 5,86 | 0,02 | 12,9 | 2,24 | 0,11 | 0,89 | 0,09 | 0,06 | 0,13 |
| 7 | 3 % | F | 48 | childhood | TCS, TCI | No | No | ARC | 697 | 5,91 | 0,11 | 0,1 | 2,2 | 0,15 | 0,24 | 6,09 | 0,65 | 0,17 |
| 8 | 10 % | F | 22 | childhood | TCS, TCI, NB-UVB | No | ARC | 0,1 | 1013 | 0,77 | 0,07 | 0,08 | 0,08 | 0,06 | 100 | 55,1 | 1,03 | 0,31 |
| 9 | 10 % | M | 39 | adolescence | TCS, TCI, systemic corticosteroids | No | No | 0,23 | 115 | 7,8 | 0,01 | 0,82 | 0,3 | 0,11 | 8,32 | 4,6 | 0,49 | 0,04 |
| 10 | 10 % | F | 18 | early childhood | TCS, TCI | No | ARC | 0,11 | 130 | 0,18 | - | - | - | - | - | - | - | - |
| 11 | 30 % | M | 26 | 4 y | TCS, TCI | No | ARC | 0,25 | 47 | 0,34 | - | - | - | - | - | - | - | - |
| 12 | 30 % | F | 37 | childhood | TCS, TCI | No | ARC | - | - | - | - | - | - | - | - | - | - | - |
| 13 | 30 % | M | 53 | adolescence | TCS, TCI, systemic corticosteroids | No | ARC, Food allergy | 1,14 | 21787 | 58,3 | 0,6 | 0,69 | 1,01 | 2,39 | 1,23 | 4,15 | 4,96 | 48,7 |

Patient characteristics, baseline eosinophil counts, total serum IgE-levels and specific IgEs to aeroallergens.

Abbreviations: F: Female, M: Male, TCS: Topical corticosteroids, TCI: Topical calcineurin inhibitors, ARC: Allergic rhinoconjunctivitis, CyA: Cyclosporin A (p.o.), MTX: methotrexate (p.o.), NB-UVB: Narrowband UVB phototherapy
