# Supplementary material for: Topical probiotic Lactobacillus lactis treatment in atopic dermatitis: a placebo-controlled pilot study on tolerability and efficacy
Source: Front Med (Lausanne). 2026 Feb 3;13:1694229. doi: 10.3389/fmed.2026.1694229 (PMC12910470; doi:10.3389/fmed.2026.1694229)
Supplement: Supplementary file 2 [file Table_2.docx]

**Supplementary material 2:**

Study protocol

Patient selection

Inclusion criteria

3%

10%

30%

Placebo

BL doctor’s examination, start of 4 weeks experimental cream

Doctor’s examination after 4 weeks of experimental cream, start of 4 weeks of follow-up

1 drop out

Doctor’s examination after 4 weeks of follow-up

1 drop out

BL

4 weeks

8 weeks

13 patients

Randomization
