# Supplementary material for: Topical probiotic Lactobacillus lactis treatment in atopic dermatitis: a placebo-controlled pilot study on tolerability and efficacy
Source: Front Med (Lausanne). 2026 Feb 3;13:1694229. doi: 10.3389/fmed.2026.1694229 (PMC12910470; doi:10.3389/fmed.2026.1694229)
Supplement: Supplementary file 3 [file Table_3.docx]

**Supplementary material 3:**

Statistical comparisons and composition of investigated probiotic cream

**Table 1:** Kruskal-Wallis *H*-test, measures of AD severity

|  | Comparison 30% vs. placebo | Comparison 10% vs. placebo | Comparison 3% vs. placebo |
| --- | --- | --- | --- |
| EASI BL (local) | 0.83 (CI: 0.79-1.00) | 0.27 (CI: 0.00-0.46) | 0.27 (CI: 0.21-0.94) |
| EASI 4 weeks (local) | 0.83 (CI: 0.79-1.00) | 0.51 (CI: 0.38-1.00) | 0.66 (CI: 0.60-1.00) |
| EASI 8 weeks (local) | 0.28 (CI: 0.12-0.65) | 0.51 (CI: 0.38-1.00) | 0.25 (CI: 0.21-0.94) |
| EASI BL (total) | 0.83 (CI: 0.79-1.00) | 1.00 (CI: 0.79-1.00) | 0.72 (CI: 0.65-1.00) |
| EASI 4 weeks (total) | 0.51 (CI: 0.35-0.88) | 0.83 (CI: 0.79-1.00) | 0.83 (CI: 0.79-1.00) |
| EASI 8 weeks (total) | 0.83 (CI: 0.79-1.00) | 0.51 (CI: 0.27-0.81) | 0.08 (CI: 0.00-0.22) |
| IGA BL | 0.82 (CI: 0.79-1.00) | 0.32 (CI: 0.05-1.00) | 0.66 (CI: 0.35-0.88) |
| IGA 4 weeks | 1.00 (CI: 0.79-1.00) | 0.32 (CI: 0.05-1.00) | 0.49 (CI: 0.27-0.81) |
| IGA 8 weeks | 0.82 (CI: 0.79-1.00) | 0.35 (CI: 0.05-1.00) | 0.14 (CI: 0.00-0.35) |
| TEWL BL (eczema) | 0.28 (CI: 0.12-0.65) | 0.83 (CI: 0.78-1.00) | 0.48 (CI: 0.27-0.81) |
| TEWL 4 weeks (eczema) | 0.83 (CI: 0.79-1.00) | 0.28 (CI: 0.14-0.70) | 0.51 (CI: 0.27-0.81) |
| TEWL 8 weeks (eczema) | 0.51 (CI: 0.44-0.94) | 0.83 (CI: 0.78-1.00) | 0.25 (CI: 0.06-0.56) |
| TEWL BL (control) | 0.05 (CI: 0.00-0.22) | 0.13 (CI: 0.00-0.38) | 0.16 (CI: 0.06-0.59) |
| TEWL 4 weeks (control) | 0.51 (CI: 0.44-0.94) | 0.50 (CI: 0.76-1.00) | 0.18 (CI: 0.00-0.46) |
| TEWL 8 weeks (control) | 0.28 (CI: 0.12-0.65) | 0.28 (CI: 0.14-0.70) | 0.08 (CI: 0.00-0.35) |

**Table 2:** Kruskal-Wallis *H*-test, QoL indicators

|  | Comparison 30% vs. placebo | Comparison 10% vs. placebo | Comparison 3% vs. placebo |
| --- | --- | --- | --- |
| Pruritus VAS BL | 0.66 (CI: 0.54-0.99) | 0.35 (CI: 0.27-0.81) | 0.86 (CI: 0.78-1.00) |
| Pruritus VAS 4 weeks | 0.51 (CI: 0.35-0.88) | 0.82 (CI: 0.79-1.00) | 0.66 (CI: 0.44-0.94) |
| Pruritus VAS 8 weeks | 0.50 (CI: 0.35-0.88) | 1.00 (CI: 0.79-1.00) | 0.08 (CI: 0.00-0.46) |
| Sleep disturbance VAS BL | 0.49 (CI: 0.35-0.88) | 1.00 (CI: 0.79-1.00) | 0.58 (CI: 0.27-0.81) |
| Sleep disturbance VAS 4 weeks | 0.82 (CI: 0.79-1.00) | 0.83 (CI: 0.79-1.00) | 0.18 (CI: 0.00-0.46) |
| Sleep disturbance VAS 8 weeks | 0.82 (CI: 0.79-1.00) | 0.83 (CI: 0.79-1.00) | 0.55 (CI: 0.44-0.94) |
| DLQI BL | 0.66 (CI: 0.35-0.88) | 0.83 (CI: 0.79-1.00) | 0.72 (CI: 0.44-0.94) |
| DLQI 4 weeks | 0.83 (CI: 0.79-1.00) | 0.83 (CI: 0.79-1.00) | 0.51 (CI: 0.27-0.81) |
| DLQI 8 weeks | 0.83 (CI: 0.79-1.00) | 0.83 (CI: 0.79-1.00) | 0.25 (CI: 0.12-0.65) |
| POEM BL | 0.83 (CI: 0.79-1.00) | 0.83 (CI: 0.79-1.00) | 0.29 (CI: 0.12-0.65) |
| POEM 4 weeks | 0.51 (CI: 0.35-0.88) | 0.83 (CI: 0.79-1.00) | 0.66 (CI: 0.65-1.00) |
| POEM 8 weeks | 0.51 (CI: 0.35-0.88) | 0.83 (CI: 0.79-1.00) | 0.14 (CI: 0.00-0.46) |
| ADCT BL | 0.66 (CI: 0.65-1.00) | 0.51 (CI: 0.35-0.88) | 0.48 (CI: 0.35-0.88) |
| ADCT 4 weeks | 0.66 (CI: 0.65-1.00) | 0.83 (CI: 0.79-1.00) | 0.83 (CI: 0.79-1.00) |
| ADCT 8 weeks | 0.83 (CI: 0.79-1.00) | 0.83 (CI: 0.79-1.00) | 0.14 (CI: 0.06-0.56) |

**Table 3:** Friedman test, measures of AD severity

|  | Comparison 30% weeks 0, 4 and 8 | Comparison 10% weeks 0, 4 and 8 | Comparison 3% weeks 0, 4 and 8 | Comparison placebo weeks 0, 4, and 8 |
| --- | --- | --- | --- | --- |
| EASI (local) | 0.31 (CI: 0.06-0.56) | 0.91 (CI: 0.79-1.00) | 0.22 (CI: 0.19-0.73) | 0.53 (CI: 0.35-0.88) |
| EASI (total) | 0.76 (CI: 0.65-1.00) | 0.72 (CI: 0.54-1.00) | 0.22 (CI: 0.06-0.56) | 0.72 (CI: 0.54-1.00) |
| IGA | 0.61 (CI: 0.37-1.00) | 0.37 (CI: N/A) | 0.61 (CI:0.55-1.00) | 0.37 (CI: N/A) |
| TEWL (eczema) | 0.26 (CI: 0.12-0.65) | 0.72 (CI: 0.65-1.00) | 0.61 (CI: 0.35-0.88) | 0.72 (CI: 0.65-1.00) |
| TEWL (control) | 0.37 (CI: 0.19-0.73) | 0.72 (CI: 0.54-1.00) | 0.16 (CI: 0.12-0.65) | 0.10 (CI: 0.06-0.56) |

**Table 4:** Friedman test, QoL indicators

|  | Comparison 30% weeks 0, 4 and 8 | Comparison 10% weeks 0, 4 and 8 | Comparison 3% weeks 0, 4 and 8 | Comparison placebo weeks 0, 4, and 8 |
| --- | --- | --- | --- | --- |
| Pruritus VAS | 0.67 (CI: 0.55-1.00) | 0.15 (CI: 0.06-0.56) | 0.22 (CI: 0.19-0.73) | 0.67 (CI: 0.44-0.94) |
| Sleep disturbance VAS | 1.00 (CI: 0.79-1.00) | 0.22 (CI: 0.10-0.70) | 0.22 (CI: 0.19-0.81) | 0.61 (CI: 0.55-1.00) |
| DLQI | 0.76 (CI: 0.65-1.00) | 0.76 (CI: 0.65-1.00) | 0.22 (CI: 0.12-0.65) | 0.15 (CI: 0.00-0.55) |
| POEM | 0.76 (CI: 0.35-0.88) | 0.76 (CI: 0.65-1.00) | 0.22 (CI: 0.00-0.46) | 0.76 (CI: 0.45-1.00) |
| ADCT | 0.72 (CI: 0.65-1.00) | 0.15 (CI: 0.00-0.46) | 0.37 (CI: 0.12-0.65 ) | 0.76 (CI: 0.55-1.00) |

**Table 5:** Components of vehicle and *L. lactis CG* probiotic lysate cream.

| Ingredients, placebo | Ingredients, experimental cream |
| --- | --- |
| Shea butter | Shea butter |
| Isopropyl palmitate | Isopropyl palmitate |
| Medium chain trigycerides | Medium chain trigycerides |
| Emulcire 61 (Cetyl alcohol, Ceteth-20, Steareth-20) | Emulcire 61 (Cetyl alcohol, Ceteth-20, Steareth-20) |
| Gelot 64 (Glyceryl stearate, PEG-75 stearate) | Gelot 64 (Glyceryl stearate, PEG-75 stearate) |
| Glycerol (85%) | Glycerol (85%) |
| 1,3-butylene glycol | 1,3-butylene glycol |
| Xantham gum | Xantham gum |
| Purified water | Purified water |
| Sodium lactate | Sodium lactate |
|  | Lactococcus lysate 3%/10%/30% |

The *L. lactis CG* lysate cream contained different concentrations Lactococcus lysate. These concentrations were 3%, 10% and 30% (different patient groups).
